# Amelioration effects against *N*-nitrosodiethylamine and CCl_4_-induced hepatocarcinogenesis in Swiss albino rats by whole plant extract of *Achyranthes aspera*

**DOI:** 10.4103/0253-7613.71921

**Published:** 2010-12

**Authors:** R. Kartik, Ch. V. Rao, S.P. Trivedi, P. Pushpangadan, G.D. Reddy

**Affiliations:** Translational Cancer Research laboratory, Rajiv Gandhi Center for Biotechnology, Thycaud P.O, Poojapura, Thiruvananthapuram - 695 014, Kerala; Pharmacognosy and Ethnopharmacology Division, National Botanical Research Institute, Rana Pratap Marg, Lucknow - 226 001; 1 Environmental Toxicology Laboratory, Department of Zoology, University of Lucknow, Lucknow - 226 007, India; 2 Amity Institute for Herbal and Biotech Products Development, Thiruvananthapuram - 695 014, India; 3 Department of Pharmacology, Central Research Institute (Ayurveda), Kolkata - 700 091, India

**Keywords:** *Achyranthes aspera*, CCl_4_, hepatic diagnostic marker, N-nitrosodiethylamine, oxidative enzymes

## Abstract

**Objective:**

The prevalence of oxidative stress may be implicated in the etiology of many pathological conditions. Protective antioxidant action imparted by many plant extracts and plant products make them a promising therapeutic drug for free-radical-induced pathologies. In this study, we assessed the antioxidant potential and suppressive effects of *Achyranthes aspera* by evaluating the hepatic diagnostic markers on chemical-induced hepatocarcinogenesis.

**Materials and Methods:**

The *in vivo* model of hepatocarcinogenesis was studied in Swiss albino rats. Experimental rats were divided into five groups: control, positive control (NDEA and CCl_4_), *A. aspera* treated (100, 200, and 400 mg/kg b.w.). At 20 weeks after the administration of NDEA and CCl_4_, treated rats received *A. aspera* extract (AAE) at a dose of 100, 200, and 400 mg/kg once daily route. At the end of 24 weeks, the liver and relative liver weight and body weight were estimated. Lipid peroxidation (LPO), superoxide dismutase (SOD), catalase (CAT), glutathione peroxidase (GPx), glutathione-S-transferase (GST), and reduced glutathione (GSH) were assayed. The hepatic diagnostic markers namely serum glutamic oxaloacetic transminase (AST), serum glutamic pyruvate transminase (ALT), serum alkaline phosphatase (ALP), gamma glutamyl transpeptidase (GGT), and bilirubin (BL) were also assayed, and the histopathological studies were investigated in control, positive control, and experimental groups.

**Results:**

The extract did not show acute toxicity and the *per se* effect of the extract showed decrease in LPO, demonstrating antioxidant potential and furthermore no change in the hepatic diagnosis markers was observed. Administration of AAE suppressed hepatic diagnostic and oxidative stress markers as revealed by decrease in NDEA and CCl_4_ -induced elevated levels of SGPT, SGOT, SALP, GGT, bilirubin, and LPO. There was also a significant elevation in the levels of SOD, CAT, GPx, GST, and GSH as observed after AAE treatment. The liver and relative liver weight were decreased after treatment with AAE in comparison to positive control group. The architecture of hepatic tissue was normalized upon treatment with extract at different dose graded at 100, 200, and 400 mg/kg. b.w. in comparison to positive control group.

**Conclusion:**

These results suggest that *A. aspera* significantly alleviate hepatic diagnostic and oxidative stress markers which signify its protective effect against NDEA and CCl^4^-induced two-stage hepatocarcinogenesis.

## Introduction

Hepatocellular carcinoma (HCC) is the fifth most common cancer worldwide with poor diagnosis and accounts for approximately 549,000 deaths each year.[1] Hepatitis infection, toxic industrial chemicals, food additives, alcohol, fungal toxins (aflatoxins), air, and water pollutants are the major risk factors of liver diseases.[2] Human liver is the major site in the body that metabolizes ingested material. It is prone to carcinogenic insult. Moreover, due to high tolerance of liver, HCC is seldom detected at the early stage and once detected treatment faces a poor prognosis in most cases.[3]

*N*-nitrosodiethylamine (NDEA) is a potent hepatocarcinogenic dialkylnitrosamine present in various foodstuffs such as milk products, meat products, soft drinks, and alcoholic beverages.[4] Mechanism of NDEA-induced carcinogenesis include DNA adduct formation followed by gene mutation, cytolethality following regenerative proliferation and oxidative stress or damage by impairment of mitochondrial respiration by free radicals.[5,6]

*Achyranthes aspera* (L.) (Family: *Amaranthaceae*) has many beneficial uses. It has been reported to possess many beneficial effects such as anticoagulant,[7] immunostimulant,[8] antiinflammatory and antiarthritis,[9] antitumor[10] as well as hypoglycemic activity.[11] Administration of *A. aspera* extract has been shown to elevate thyroid hormone levels and decreases hepatic lipid peroxidation in male rats.[12] The study was done in order to validate the inhibitory properties of *A. aspera* against *N*-nitrosodiethylamine (NDEA) and carbontetrachloride (CCl_4_)-induced hepatocarcinogenesis in rats.

## Materials and Methods

### Chemicals

Chemicals used in this study including NDEA and CCl_4_ were procured from Sigma Chemicals, St. Louis, USA, and all others chemicals were purchased from SD Fine Chemicals Ltd., Mumbai, India with highest purity grade.

### Plant material and preparation of extracts

The plant of *A. aspera* was collected, identified, and authenticated taxonomically at National Botanical Research Institute, Lucknow, India, and the voucher specimens (NAB 200494) were deposited in the departmental herbarium for future reference. The plants were washed with distilled water to remove dirt and soil, shade dried, and finely powdered. The powdered material (1000 g) was extracted thrice with 50% ethanol (v/v). The extracts were filtered, pooled, and concentrated at 50°C on a rotary evaporator (Buchi, USA) and then freeze-dried (Freezone^®^ 4.5, Labconco, USA) at high vacuum (133 × 10^−3^ mbar) and low temperature −40 ± 2° C (yield 7.5%, wt/wt). Then, 50% ethanol extract of *A. aspera* (AAE) was stored at 4–8°C and resuspended in double distilled water containing 1% carboxymethylcellulose (CMC, w/v) at the time of administration.

### Animals

Swiss albino rats, weighing 140–160 g, were procured from the National Laboratory Animal Centre (NLAC), Central Drug Research Institute, Lucknow, India. They were kept in the departmental animal house for 1 week before and during the experiments, in cross-ventilated room at 27 ± 2°C with relative humidity of 44–56%, light and dark cycles of 10 and 14 h, respectively. Animals were fed on standard rodent pellet diet (Amrut, Lucknow, India) and food was withdrawn 18–24 h prior to though water was allowed *ad libitum*. All experiments conducted were in accordance with the Institutional Ethical Committee and the Institutional Animal Ethics Committee, CPCSEA, India (Reg. No. 222/2000/CPCSEA).

### Acute toxicity study

The acute toxicity of 50% ethanol extract of *A. aspera* (AAE) was evaluated in mice using the up and down procedure. Mice of either sex (three females and three males, weight: 25–35 g, age: 6–8 weeks) received AAE starting at 2 g/kg orally by gavage. The animals were observed for toxic symptoms continuously for the first 4 h after dosing. The number of survivors were noted after 24 h, and these animals were then maintained and observed daily for next 13 days for any further toxicity.

### In vivo study on NDEA and CCl_4_-induced hepatocellular carcinoma in Swiss albino rat

Five groups of 6 Swiss albino rats each were included in this experiment. Groups I and II were normal/placebo control and positive control groups, respectively, while groups III, IV, and V were treated groups. All the groups except group I were administered NDEA (200 mg/kg b.w., i.p.) followed by CCl_4_ (3 ml/kg b.w., s.c.) once a week for 6 weeks as described.[13] After 20 weeks of postexposure to NDEA and CCl_4_, treated groups were administered orally once daily with 100, 200, and 400 mg/kg b.w. of AAE in CMC (vehicle) for 4 consecutive weeks. The groups II and I received CMC (1 ml/kg, p.o.). The standard orogastric cannula was used for oral administration. At the end of 24 weeks, all the rats were killed by cervical dislocation after an overnight fasting and the blood were collected to assess the levels of hepatic diagnostic markers and the liver for histopathological and antioxidant enzymes level.

### Biochemical analysis

Serum transaminases (AST and ALT) were determined by the method of Reitman and Frankel.[14] The alkaline phosphatase levels were estimated by method of King and Armstrong.[15] The gamma glutamyl transpeptidase (GGT) activity was determined according to the method of Szas.[16] The bilirubin level in serum was determined by modified DMSO method of Walters and Gerarde.[17]

### Enzyme assay

Hepatic tissues were homogenized in KCl (10 mM) phosphate buffer (1.15%) with ethylene-diamine tetra acetic acid (EDTA; pH 7.4) and centrifuged at 12,000 × g for 60 min. The supernatant were used for glutathione peroxidase (*GPx*), glutathione-S-transferase (GST), superoxide dismutase, catalase, reduced glutathione (GSH), and thiobarbituric acid reactive substances (TBARS) estimation. The concentration of TBARS were measured (lipid peroxidation product maondialdehyde (MDA) was estimated) in liver by the method of Ohkawa *et al*.[18] SOD was estimated according to the method of Kakkar *et al*.[19] CAT was estimated by the method of Aebi.[20] The enzyme GST was measured according to the method of Habig and Jacoby.[21] Reduced glutathione was measured by the method of Ellman.[22] Glutathione peroxidase was measured by the method of Rotruck *et al*.[23]

### Histopathological study

Histopathological studies were performed as per the standard protocol of Luna.[24]

### Statistical analysis

Data are expressed as mean ± SEM (standard error of mean). The difference among means has been analysed by unpaired Student’s *t*-test.

## Results

### Toxicity study

Over the study duration of 14 days, there were no deaths recorded in the male and female animals, given 2 g/kg of the 50% ethanolic extract of *A. aspera* orally. During the observation period, animals did not produce any variations in the general appearance. The acute toxicity study does not show any toxic symptoms, changes in behavior, or mortality at 2 g/kg doses. All animals survived until the scheduled euthanasia and no gross pathological alteration was found in the internal organs.

### Per se effect of the 50% ethanolic extract of *A. aspera* on SGOT, SGPT, SALP, and bilirubin level (BL) in serum

The 50% ethanolic extracts of *A. aspera* at a dose of 100, 200, and 400 mg (OD × 28 days) were subjected for per se effect by studying various liver biochemical marker parameters such as SGOT, SGPT, SALP, and BL. However, the 50% ethanolic extracts of *A. aspera* did not show any abnormal increase in the level of SGOT, SGPT, SALP, and BL as presented in Table 1.

**Table 1:** Effect of the 50% ethanolic extract of *A. aspera* (100, 200, and 400 mg) on SGOT (U/l), SGPT (U/l), SALP (U/l), and bilirubin (BL) level (U/l) in serum of rat

*Oral treatment (mg/kg, OD × 28 days)*	*SGOT*	*SGPT*	*SALP*	*BL*
Control	198.0 ± 1.53	84.5 ± 1.10	234.1 ± 1.09	0.75 ± 0.04
*A. aspera*, 100 mg	198.1 ± 1.55	84.2 ± 1.13	234.3 ± 1.20	0.74 ± 0.03
*A. aspera*, 200 mg	197.6 ± 1.51	84.6 ± 1.52	233.8 ± 1.23	0.69 ± 0.02
*A. aspera*, 400 mg	197.8 ± 1.50	84.2 ± 1.04	239.8 ± 1.18	0.76 ± 0.04

Values are mean ± SEM of eight rats in each group.

### Per se effect of the 50% ethanolic extract of *A. aspera* on lipid peroxidation (LPO), superoxide dismutase (SOD), catalase (CAT), and glutathione peroxidase (*GPx*)

The 50% ethanolic extracts of *A. aspera* at a dose of 100, 200, and 400 mg (OD × 28 days) were subjected for per se effect by studying LPO, SOD, CAT, and GPX in liver homogenate of rats. A significant decrease (45.4, 31.8, and 13.6%) in the level of lipid peroxidation product malondialdehyde (LPO) at a dose of 400, 200, and 100 mg/kg b.w. and a significant increase in the level of SOD (31.7, 28.1, and 13.8%), CAT (27.2, 19.1, and 10.7%) at 400, 200 and 100 mg/kg b.w was observed. GPx also showed increase at 400 mg/kg b.w. (28.1%) and 200 mg/kg b.w. (6.2%) of AAE-treated animals compared to the control animals as shown in Table 2.

**Table 2:** Effect of the 50% ethanolic extract of *A. aspera* (100, 200, and 400 mg) on lipid peroxidation (LPO, MDA nmoles/mg of protein), superoxide dismutase (SOD, units/mg of protein), catalase, (CAT, units/mg of protein), and glutathione peroxidase (GPx, mmol/g tissue) in rats

*Oral treatment (mg/kg, OD × 28 days)*	*LPO*	*SOD*	*Catalase*	*GPx*
Control	0.44 ± 0.02	110.3 ± 9.5	26.1 ± 1.4	3.2 ± 0.04
*A. aspera*, 100 mg	0.38 ± 0.02	125.6 ± 8.8	28.9 ± 1.5	3.2 ± 0.05
*A. aspera*, 200 mg	0.30 ± 0.01***	141.0 ± 9.1*	31.1 ± 1.6*	3.4 ± 0.05**
*A. aspera*, 400 mg	0.24 ± 0.02***	144.9 ± 11.9*	33.2 ± 1.2**	4.1 ± 0.07***

Values are mean ± SEM of eight rats in each group. Values not sharing common superscript letter differ significantly from control group basal level and

* *P* < 0.05

** *P* < 0.01

*** *P* < 0.001 compared with control group.

### Body weight (initial and final), mean and relative liver weight

A significant increase in mean liver weight (50%) and increase in relative liver weight (90.6%) in positive control group with respect to control group was observed. It was also found that treated group receiving a dose of 100, 200, and 400 mg/kg of AAE showed a dose-dependent reduction in the mean liver weights of the carcinogenic groups (15.9, 24.6, and 33.3%) and also the relative liver weights (31.1, 39.3, and 44.2%), respectively, as shown in Table 3. In comparison to final body weight of normal group of rats, a significant decrease (23.8%) in positive control group following NDEA treatment was observed. In AAE-treated groups, the final bodyweight showed gain (20.1, 26.1, and 28.3%) at a dose graded of 100, 200, and 400 mg/kg, respectively.

**Table 3:** Effect of 50% ethanolic extract of *A. aspera* on body weight, liver weight, and relative liver weight in (NDEA +CCl4) induced HCC rat

*Treatment*	*Dose*	*Initial body weight (g)*	*Final body weight (g)*	*Liver weight (g)*	*Relative liver weight (liver weight/100 g b.w.)*
Control (CMC)	1mL/kg	141 ± 8	176 ± 8	4.6 ± 0.31	3.2 ± 0.44
NDEA + CCl_4_	200 mg/kg (NDEA) + 3 ml/kg b.w. (CCl_4_)	154 ± 9	134 ± 9	6.9 ± 0.72***	6.1 ± 1.14
*A. aspera*	100 mg/kg	153 ± 7	161± 8	5.8 ± 0.39	4.2 ± 0.64
*A. aspera*	200 mg/kg	158 ± 7	169 ± 7	5.2 ± 0.43	3.7 ± 0.41
*A. aspera*	400 mg/kg	145 ± 6	172 ± 7	4.6 ± 0.58*	3.4 ± 0.21

Values are mean ± SEM of six rats in each group. Values not sharing common superscript letter differ significantly from control group basal level and

*** *P* < 0.001 compared with respective control group.

* *P* < 0.05 as compared with group (NDEA + CCl_4_).

### Biochemical analysis

It is clearly evident that in positive control group (NDEA and CCl_4_) caused significant elevation (*P* < 0.001) in the levels of hepatic markers such as SGOT, SGPT, SALP, and GGT in comparison with control group as shown in Table 4. In AAE-treated groups at different doses of 100, 200, and 400 mg/kg dose-dependent reduction in the levels of hepatic markers was observed. The range of hepatic markers was found to be statistically significant at different graded dose-dependent manner with respect to the positive control group.

**Table 4:** Effect of the 50% ethanolic extract of *A. aspera* on SGOT (U/l), SGPT (U/l), SALP (U/l), bilirubin level (U/l), and gamma glutamyl transpeptidase (GGT) (U/l) in serum of rat

*Treatment*	*Dose*	*SGOT*	*SGPT*	*SALP*	*BL*	*GGT*
Control (CMC)	1 mL/kg	191.22 ± 3.19	81.25 ± 1.58	231.11 ± 11.31	0.76 ± 0.03	30.8 ± 5.1
NDEA + CCl_4_	200 mg/kg (NDEA) ± 3 ml/kg b.w. (CCl_4_)	362.21 ± 22.32^***f^	378.51 ± 29.78^***f^	437.38 ± 28.33^***f^	1.32 ± 0.06^***f^	158.9 ± 8.2^***f^
*A. aspera*	100 mg/kg	294.14 ± 19.51*	325.33 ± 27.24	348.22 ± 24.38*	1.18 ± 0.05	142.1± 6.8
*A. aspera*	200 mg/kg	253.48 ± 18.36**	218.11 ± 26.21**	284.21 ± 23.33**	0.89 ± 0.13*	124.8 ± 5.6**
*A. aspera*	400 mg/kg	218.84 ± 18.90***	124.03 ± 22.31***	243.91 ± 21.83***	0.81 ± 0.12**	74.9± 4.2***

Values are mean ± SEM of six rats in each group. Values not sharing common superscript letter differ significantly from control group basal level and ***P < 0.001 compared with respective control group and the superscript letter “f” indicates a significant difference (***P < 0.001) between group (NDEA + CCl_4_) and control group.

* *P* < 0.05.

** *P* < 0.01.

*** *P* < 0.001 compared with group (NDEA + CCl_4_).

### Antioxidant enzyme assays

The levels of different oxidative markers such as LPO, SOD, CAT, GST, GSH, and GPx were analyzed *prior* to administration of the AAE in positive control group and compared with the control group. Except the LPO levels, the levels of SOD, CAT, GST, GSH, and GPx were significantly reduced (*P* < 0.001) in the positive control group with respect to the controls. The LPO levels, on the other hand, were found to be significantly increased (*P* < 0.001) in the positive control group as compared with the control group as shown in Table 5. After AAE treatment in the NDEA and CCl_4_-induced group, significant increase in the levels of all the antioxidant enzymes were noted. At 400 mg/kg b.w., the AAE significantly normalized to a significant level in LPO (*P* < 0.01), SOD (*P* < 0.001), CAT (*P* < 0.001), GST (*P* < 0.001), and GPx (*P* < 0.001) and in GSH. A dose-graded response was also observed in the enzymes administered at 100 and 200 mg/kg, b.w., respectively.

**Table 5:** Effect of 50% ethanolic extract of *A. aspera* on liver superoxide dismutase (SOD, units/mg of protein), catalase (CAT, units/mg of protein), lipid peroxidation (LPO, MDA nmoles/mg of protein), glutathione peroxidase (GPx, μg/mg), glutathione-S-transferase (GST, μg/mg of protein) and reduced glutathione (GSH, μg/mg of protein)

*Treatment*	*Dose*	*SOD*	*CAT*	*LPO*	*GPx*	*GST*	*GSH*
Control (CMC)	1 mL/kg	116.12 ± 8.4	26.02 ± 1.60	0.56 ± 0.01	4.58 ± 0.02	1.14 ± 0.16	3.48 ± 0.34
NDEA + CCl_4_	200 mg/kg (NDEA) + 3 ml/kg b.w. (CCl_4_)	37.87 ± 8.12***f	7.38 ± 0.61***f	6.04 ± 1.24***f	1.40 ± 0.01***f	0.49 ± 0.02***f	3.06 ± 0.32
*A. aspera*	100 mg/kg	62.18 ± 9.56	11.67 ± 1.40*	4.89 ± 0.43	2.67 ± 0.02***	0.76 ± 0.04***	3.19 ± 0.34
*A. aspera*	200 mg/kg	81.29 ± 12.49*	14.38 ± 1.32***	2.24 ± 0.54*	2.92 ± 0.03***	0.84 ± 0.02***	3.21 ± 0.35
*A. aspera*	400 mg/kg	92.50 ± 9.21***	18.89 ± 0.89***	1.19 ± 0.82**	3.12 ± 0.02***	0.98 ± 0.05***	3.31 ± 0.32

Values are mean ± SEM of six rats in each group. Values not sharing common superscript letter differ significantly from control group basal level and ***P < 0.001 compared with respective control group and the superscript letter “f” indicates a significant difference (***P < 0.001) between group (NDEA + CCl_4_) and control group.

* *P* < 0.05.

** *P* < 0.01.

*** *P* < 0.001 compared with group (NDEA + CCl_4_).

### Histopathology study

**Figure 1:**
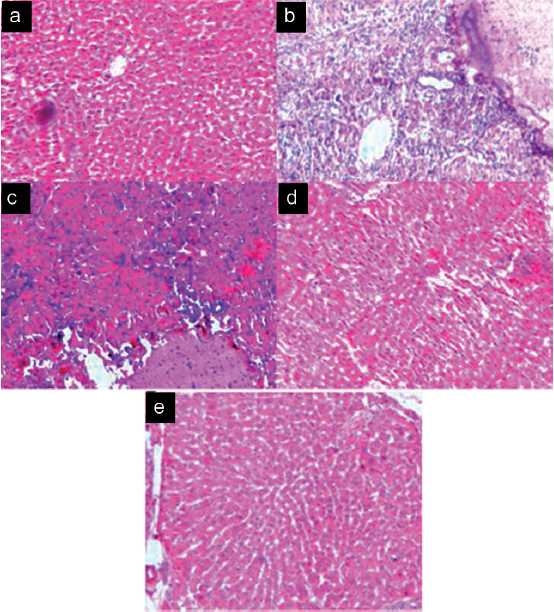
Effect of 50% ethanolic extract of *Achyranthes aspera* on NDEA and CCl4 induced HCC. (a) Group I: Control animals show normal architecture. (b) Group II: NDEA + CCL4 induced cancer bearing animal shows neoplastic cells are arranged in lobules. (c) Group III: Administration of 50% ethanolic extract (100 mg/kg) of *A. aspera* (whole plant) on HCC bearing animals. Necrosis with malignant hepatocytes. (d) Group IV: Administration of 50% ethanolic extract (200 mg/kg) of *A. aspera* (whole plant) on HCC bearing animals. Liver shows the structure close to proximity of normal liver. (e) Group V: Administration of 50% ethanolic extract (400 mg/kg) of *A. aspera* (whole plant) on HCC bearing animals. Liver shows normal architecture of hepatocytes.

Histopathological observations were found to support the findings of serum tumor markers analysis as shown in Figure 1. The control group of animals showed normal histological liver architecture having cells with granulated cytoplasm and small uniform nuclei [Figure 1A]. On the other hand, the positive control group showed significant loss of liver architecture. This figure shows significant tumor thrombi in both hepatic and portal vessels. The histologic appearance of HCC is also extremely variegated. The tumor cells are seen to grow in nests and thick cords and are separated from one another by thin-walled sinusoids. Cytologically, the tumor cells bear some resemblance to normal hepatocytes, but are slightly larger, have more irregular and prominent nuclei [Figure 1B]. In contrast the group treated with AAE at a dose of 100 mg/kg, b.w. showed necrosis with malignant hepatocytes [Figure 1C]. The group treated with AAE at a dose of 200 mg/kg, b.w. showed well-defined structures and hepatocytes maintaining near normal architecture [Figure 1D], whereas in the group treated with 400 mg/kg. b.w. of AAE showed normal hepatocyte architecture with well-defined aggregation of hepatic and portal veins [Figure 1E]. The potential beneficial effects of AAE on the liver tissue were also visible evident from histological findings. Histological examination also showed substantial improvement in the overall tissue architecture.

## Discussion

The toxicological studies of *A. aspera* were conducted for their safety and toxicity. The plant extracts were subjected to preliminary acute toxicity study in mice at different dose levels. The results showed no abnormal symptoms or any mortality in the test animals. In this study, the 50% ethanolic extracts of *A. aspera* per se showed dose-dependent antioxidant activity as evidenced by their effects on elevated levels of SOD, CAT, GPX, and depleted levels of LPO. Furthermore, there were no changes observed in the biochemical markers namely, SGOT, SGPT, and ALP. This indicates that *A. aspera* extract contributes to exert antioxidant defense mechanism by metabolizing lipid peroxides and scavenging endogenous peroxides.

The activity of AAE was also evident as a direct physiological effect on liver tissue. NDEA-induced proliferation of cells in the liver tissue was evident from the increase in liver weights. The AAE markedly reduced the mean and relative liver weights as compared to the positive control group, which signify the amelioration capacity of extract upon carcinogen exposure. The pathological changes were monitored by determining the levels of various biochemical hepatic markers.[25] The rise in their levels was shown to have a good correlation with the number of transformed cells in cancer conditions. We observed significant increase in levels of SGOT, SGPT, SALP, and GGT in the positive control group with respect to those of control group. However, the AAE-treated group showed significant effect on the levels of hepatic markers to near normal level. Serum bilirubin, which is a biomarker for liver damage, is a intracellular enzyme present abundantly in the liver under normal conditions. In the case of hepatocellular damage caused by xenobiotics and carcinogenic in these enzymes leak out from the damaged hepatocytes, causing an increase in serum enzyme. In positive control group, there was an elevation in level of serum bilirubin which may be due to the leakage of plasma membrane and loss of functional integrity of cell membranes in liver. In groups treated with 50% ethanol extract (AAE) at different graded levels at 100, 200, and 400 mg/kg, reduction in the level of serum bilirubin was observed in a dose-dependent manner which indicates the restoration capacity of serum marker enzymes back to normal. The extract, thus, neutralizes the effects of NDEA-induced proliferation of cells thus suppressing carcinogenesis.

Free radical damage and oxidative stress are the major reasons for liver tissue damage that can progress to develop tumor and thus result in HCC. The antioxidant enzymes are therefore the first-line defense against such damage and thus provide protection against the deteriorating outcome.[26] Lipid peroxidation is regarded as one of the basic mechanisms of tissue damage caused by free radicals.[27,28] Administration of NDEA has been reported to generate LPO products in general. LPO can be prevented at the initiation stage by free radical scavengers and antioxidants. The observation suggests that AAE-treated group has a potential to significantly reduce the levels of LPO in a graded dose manner as compared to the positive controls. Other enzymatic and nonenzymatic antioxidants such as SOD, CAT, GPx, and GSH that were investigated are known to reduce the oxidative stress by reducing the production and accumulation of superoxide radicals (O_2_^-^).[29] GSTs are a family of detoxification enzymes involved in protecting the cells against cytotoxicity and carcinogenic chemicals by conjugating with GSH. Depletion in the activity of these antioxidant enzymes was observed in positive control group. Interestingly, AAE-treated group showed significant increase in the level of GST in grade dose level at 100, 200, and 400 mg/kg b.w., respectively. It is probable that the various phytoconstituents of the plant are involved in scavenging the free radicals from the tissues, thus, reducing oxidative stress. This, in turn, acts together for normalizing the levels of the antioxidant enzymes taken under study. The protective effect of *A. aspera* was also assessed by studying the histopathology of liver tissue. In this study, noticeable changes were observed in the architecture of liver in HCC bearing animals. These indicate the presence of neoplastic conditions following NDEA and CCl_4_ administration. In animals treated with AAE (100, 200, and 400 mg/kg b.w.), the liver architecture was preserved NDEA and CCl_4_–damage was recovered. Hence, the regression of the tumors in liver may be due to the protective effect of *A. aspera*.

Our present study indicates that the extracts of *A. aspera* possess antioxidant properties and could serve as free radical inhibitors or scavenger or acting possibly as primary antioxidants. The decline in the hepatic marker shows the hepatoprotective properties of *A. aspera* against chemically (NDEA and CCl_4_) induced HCC. Recently, a lot of attention is being devoted to natural sources of antioxidant properties; the data obtained in this study might suggest a possible use of *A. aspera* as a source of natural antioxidant and antitumor agents.
